# Tree-based survival analysis improves mortality prediction in cardiac surgery

**DOI:** 10.3389/fcvm.2023.1211600

**Published:** 2023-07-10

**Authors:** Jahan C. Penny-Dimri, Christoph Bergmeir, Christopher M. Reid, Jenni Williams-Spence, Luke A. Perry, Julian A. Smith

**Affiliations:** ^1^ Department of Surgery, School of Clinical Sciences at Monash Health, Monash University, Melbourne, Australia; ^2^ Department of Data Science and Artificial Intelligence, Faculty of Information Technology, Monash University, Melbourne, Australia; ^3^ Department of Computer Science and Artificial Intelligence, University of Granada, Melbourne, Spain; ^4^ Department of Epidemiology and Preventive Medicine, Monash University, Melbourne, Vic, Australia; ^5^ Department of Anaesthesia, Victorian Heart Hospital, Monash Health, Clayton, Vic, Australia

**Keywords:** machine learning, tree-based machine learning, cardiac surgery, mortality, survival analaysis

## Abstract

**Objectives:**

Machine learning (ML) classification tools are known to accurately predict many cardiac surgical outcomes. A novel approach, ML-based survival analysis, remains unstudied for predicting mortality after cardiac surgery. We aimed to benchmark performance, as measured by the concordance index (C-index), of tree-based survival models against Cox proportional hazards (CPH) modeling and explore risk factors using the best-performing model.

**Methods:**

144,536 patients with 147,301 surgery events from the Australian and New Zealand Society of Cardiac and Thoracic Surgeons (ANZSCTS) national database were used to train and validate models. Univariate analysis was performed using Student's T-test for continuous variables, Chi-squared test for categorical variables, and stratified Kaplan-Meier estimation of the survival function. Three ML models were tested, a decision tree (DT), random forest (RF), and gradient boosting machine (GBM). Hyperparameter tuning was performed using a Bayesian search strategy. Performance was assessed using 2-fold cross-validation repeated 5 times.

**Results:**

The highest performing model was the GBM with a C-index of 0.803 (0.002), followed by RF with 0.791 (0.003), DT with 0.729 (0.014), and finally CPH with 0.596 (0.042). The 5 most predictive features were age, type of procedure, length of hospital stay, drain output in the first 4 h (ml), and inotrope use greater than 4 h postoperatively.

**Conclusion:**

Tree-based learning for survival analysis is a non-parametric and performant alternative to CPH modeling. GBMs offer interpretable modeling of non-linear relationships, promising to expose the most relevant risk factors and uncover new questions to guide future research.

## Introduction

The extraordinary physiologically stress of cardiac surgery carries a high risk of adverse postoperative outcomes (1). An important component of surgical decision-making is determining which patients will ultimately benefit after overcoming the initial insult of surgery. Overall survival is an important, albeit complex, metric for understanding the overall benefit of a surgery (2, 3). It is a layered outcome that captures the impact of peri-procedural complications and the new functional baseline achieved after the operation (2, 3). Developing interpretable tools that map a patient's physiological, operative, and early postoperative variables to their long term survival could provide novel insights into who truly benefits most from cardiac surgery.

Previous ML research modeling survival converts time-to-event to a binary outcome such as 5-year mortality, which sacrifices potentially useful information (4). Survival data is typically studied using linear models such as Cox proportional hazards (CPH) regression (5, 6). It is increasingly recognized, however, that surgical risk is non-linear (7). Novel machine learning (ML) approaches continue to be developed for survival analysis with potentially attractive non-linear properties (8, 9). Modeling relationships between variables in this way could uncover unique risk factors, assist decision-making, and improve the delivery of care.

Many important ML concepts have been translated into a survival analysis domain including neural networks, support vector machines, gradient boosting machines (GBM), and random forests (RF) (8, 10). In the context of tabular datasets, tree-based methods such as gradient boosting and RF consistently outperform deep learning methods (11). Tree-based methods tend to be more robust to uninformative features and learn non-smooth functions (11). Survival analysis in healthcare is a tabular dataset problem and therefore tree-based machine learning may provide better model fit and feature explanations.

Tree-based learners are a class of models which expand on the decision tree, whereby strong models are constructed from ensembles of weak decision trees such as the random forest (8, 12). Additionally, in gradient boosting, the optimization of the ensemble model can be improved by minimizing a residual term using the gradient of the error of the weak learner (9). Regardless of the tree-based model, survival and hazard functions are then estimated using non-parametric methods based on the data in the terminal nodes (13).

## Aims and hypotheses

Firstly, we aimed to compare model fit of tree-based machine learning to Cox proportional hazards modeling. Secondly, we aimed to use the best-performing model to determine the key predictor variables for long-term mortality. We hypothesized that tree-based ML provides better model fit and explanations compared with CPH modeling.

## Methods

### Study population

The Australian and New Zealand Society of Cardiac and Thoracic Surgeons (ANZSCTS) Database registry recorded 153,944 cardiac surgery events in 151,089 unique patients from April 2001 to December 2019, captured at 42 centers in Australia (14). The database is not publicly available as it stores sensitive patient information. Inclusion in the database was for any patient undergoing cardiac surgery, other thoracic surgery using cardiopulmonary bypass, and pericardiectomy for constrictive pericarditis, regardless of cardiopulmonary bypass. The dataset includes a linkage with the National Death Index (NDI), which is a national program recording all deaths that have occurred since 1980 (15).

### Outcome definitions

Long-term mortality was defined as any death that was recorded after a procedure. Patients were followed from the date of surgery to their death as recorded in either the database or the NDI. The date of the last linkage with the NDI, the “follow-up time”, was the 1st of August, 2019.

### Variable selection, data preparation, exploration, and statistical methodology

All perioperative variables in the database were considered for inclusion. Any variables which were missing 90% or more data were excluded from the analysis. Preliminary data analysis was conducted by comparing the univariate distribution of each variable between survivors and non-survivors at the last follow-up. Hypothesis testing for distributional differences were performed using a student's *T*-test for continuous variables and a Chi-Squared test for categorical variables. For fitting models, non-binary categorical variables were converted to a set of dummy variables.

A Kaplan-Meier (KM) estimator was fitted to plot the survival function in order to visualize the univariate effects of sex, indigenous status, and type of operation on long-term survival.

### Models

Time-to-event regression differs from standard regression as for a subset of cases the time of an event occurring has not been observed, and is thus censored. Rather than learning a direct relationship between input variables and the time of an event, survival analysis seeks to estimate the survival and cumulative hazard functions based on input covariates.

#### Cox proportional hazards regression

One of the oldest and most widely used methods for regressing censored data is the CPH model (6, 13). It uses a semi-parametric approach to learn the effects of covariates on the hazard function (6). The assumption of proportional hazards derives from the relationship between the baseline hazard function and the covariates and requires that the ratio between two patients hazard function is constant across time (6). The potential of co-linearity to degrade the performance of this model was addressed by developing a pipeline that filtered the input dataset to remove co-linearity. This was achieved by checking the correlation coefficient between features and if the coefficient exceeded 0.6 then only the feature with the highest mutual information with respect to mortality was included (16).

#### Tree-based learners

Tree-based machine learning incorporates decision tree and ensemble decision tree methods. These models are non-parametric and make no proportional hazards assumptions.

##### Decision tree

Survival trees are simple models that learn decision rules derived from input features, that is often represented as an expanding set of branching paths (17). These models are non-parametric, simple to understand, and computationally efficient (17).

##### Random forest

Random survival forests are ensembles of decision trees that are trained on bootstrapped samples of training data and a random subset of input variables (12, 13). Unlike decision trees, however, RF models do not have a simple interpretation to explain their predictions. Methods do exist, however, to determine the most important features (18).

##### Gradient boosting machine

Similar to random forests, gradient boosting machines are an ensemble of decision trees, however, the model fits an additional term to minimize the residual error of the weak learner using the gradient of the error (13). Survival and hazard functions are estimated using non-parametric methods (13).

### Training, benchmarking, and bias

For each candidate model, a hyper-parameter search was conducted using a Bayesian search strategy (19). The parameters and ranges searched are available in the supplementary materials.

**Figure 1:**
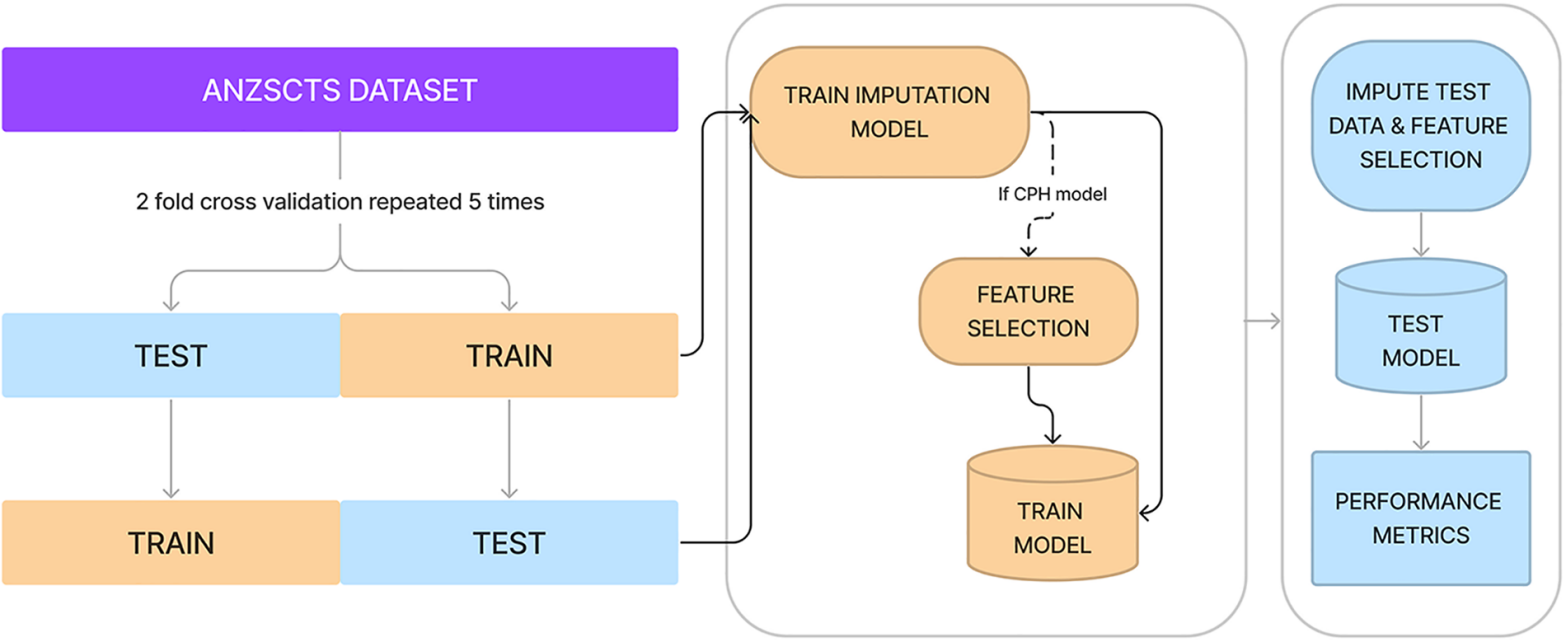
Diagrammatic representation of cross-validation, data imputation, feature selection, and measuring performance. Processes in yellow represent actions on or using the training set, whereas processes in blue are with the test set.

Performance was measured using the Concordance index (C-index) which is measured on a scale between 0.5 and 1.0, where 1.0 indicated perfect model fit and 0.5 indicates performance no better than random chance. In order to benchmark the algorithms' performance, a 2-fold cross-validation scheme repeated 5 times was used (20). Imbalanced data, where an extreme minority of samples had very long-term outcomes recorded, was handled with stratified minority class oversampling. Multiple imputation with random forests was used to impute missing data. Both imputation and oversampling were applied only to the training set of each cross-validation fold to ensure no data leak across the training and test sets. A schematic representation of training and benchmarking is presented in Figure 1.

The assessment of machine learning bias was achieved by stratifying the test set of each cross-validation fold by important characteristics and measuring the performance on the stratified subsets. Bias was assessed for the characteristics of sex and indigenous status.

### Feature importance

After benchmarking performance, the best-performing model was used to assess which features were the most important for predicting the hazard function for long-term mortality (18).

### Ethics

This project was conducted in concordance with the National Health and Medical Research Council (NHMRC) National Statement on Ethical Conduct in Human Research, with approval from the Monash University Human Research Ethics Committee (HREC) approval number 2020-24850-45439.

## Results

**Figure 2:**
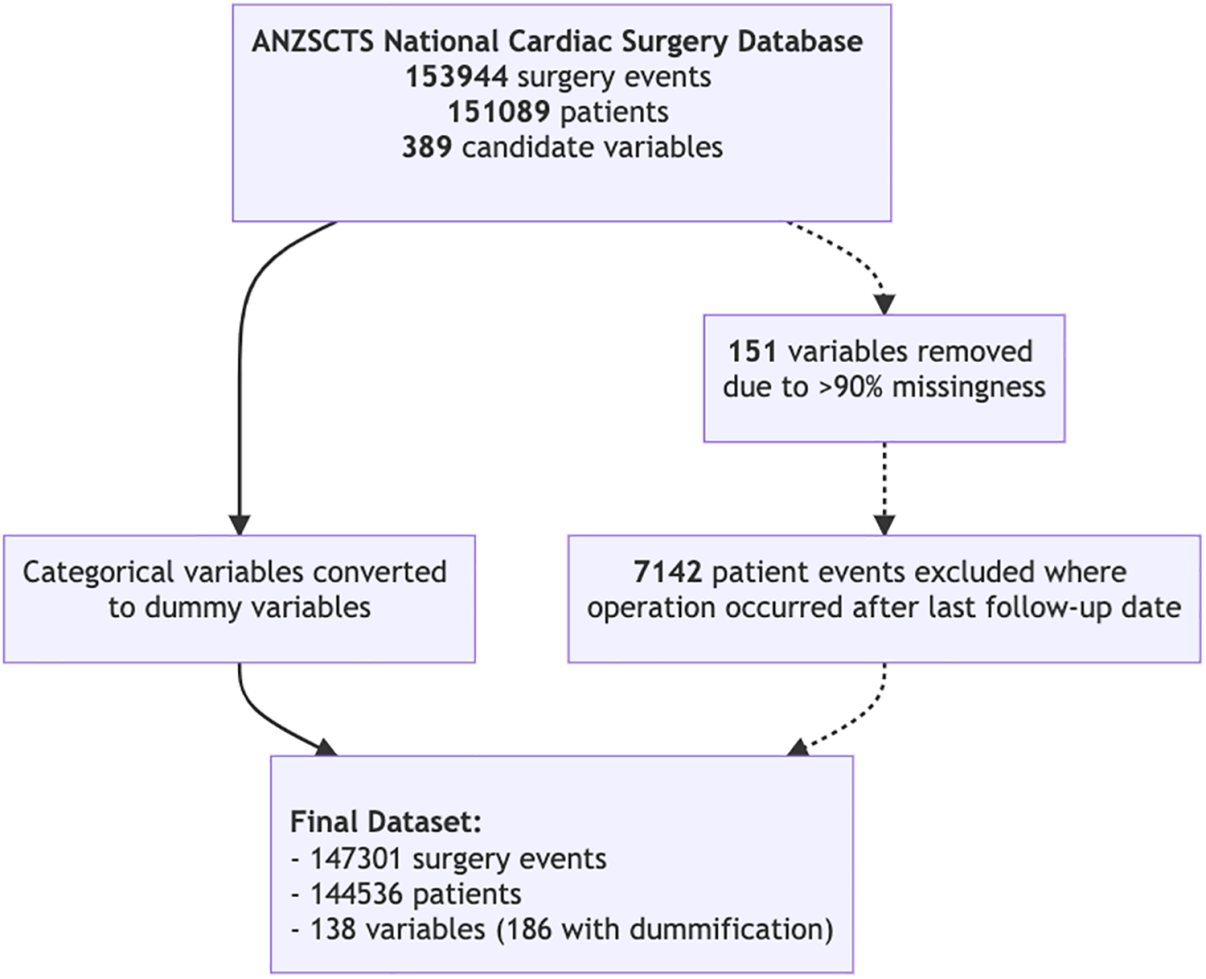
Flow diagram depicting data processing and the final subset.

144,536 patients were included in the final analysis with 147,301 surgery events (Figure 2). A summary of patient characteristics, stratified by survival at follow-up, are reported in Table 1. All patient characteristics included in the analysis are reported in supplementary Tables S1 and S2. The average age of the cohort was 65.6 years (SD 12.9), 26.8% of patients were female, and 2.6% of the cohort was indigenous. Mortality events represented 24.37% of the cohort with the longest recorded mortality 17.9 years after the first operation, and the longest survivor at follow-up was 18.2 years.

**Table 1:** Patient characteristics.

Variable	Survived (*n* = 123,750): Mean (SD) or Count (%)	Mortality (*n* = 30,194): Mean (SD) or Count (%)	*p*-value
Age	64.28 (12.87)	71.05 (11.48)	<0.001
**Sex**			
Male	91,839 (74.21%)	20,844 (69.03%)	<0.001
Female	31,911 (25.79%)	9,350 (30.97%)	
BMI	28.77 (8.03)	28.25 (9.67)	<0.001
**Indigenous**			
No	118,921 (96.10%)	29,206 (96.73%)	<0.001
Yes	3,325 (2.69%)	665 (2.20%)	
**Smoking History**			
Yes	69,496 (56.16%)	18,646 (61.75%)	<0.001
No	52,670 (42.56%)	11,258 (37.29%)	
**Diabetes**			
No	89,276 (72.14%)	19,552 (64.75%)	<0.001
Yes	34,295 (27.71%)	10,557 (34.96%)	
**Preoperative Arrhythmia**			
No	104,539 (84.48%)	22,727 (75.27%)	<0.001
Yes	19,013 (15.36%)	7,376 (24.43%)	
**Congestive Heart Failure**			
No	101,910 (82.35%)	19,511 (64.62%)	<0.001
Yes	21,663 (17.51%)	10,602 (35.11%)	
**NYHA Class**			
I	48,427 (39.13%)	9,195 (30.45%)	<0.001
II	43,970 (35.53%)	8,528 (28.24%)	
III	23,351 (18.87%)	7,911 (26.20%)	
IV	5,292 (4.28%)	3,236 (10.72%)	
Ejection Fraction	56.19 (29.53)	51.19 (15.05)	<0.001
Length of ICU Stay (Hours)	64.59 (91.89)	102.50 (186.05)	<0.001
Length of Intubation (Hours)	19.30 (59.69)	42.75 (125.92)	<0.001
**Type of Procedure**			
Isolated CABG	67,880 (54.85%)	14,290 (47.33%)	<0.001
Valve(s) only	25,429 (20.55%)	6,040 (20.00%)	
Other	19,594 (15.83%)	4,996 (16.55%)	
Valve(s) + CABG	10,690 (8.64%)	4,797 (15.89%)	
**Infective Endocarditis**			
No	120,143 (97.09%)	29,026 (96.13%)	<0.001
Yes	3,420 (2.76%)	1,075 (3.56%)	
**Urgency**			
Elective	87,018 (70.32%)	18,707 (61.96%)	<0.001
Urgent	31,667 (25.59%)	9,127 (30.23%)	
Emergency	4,755 (3.84%)	2,033 (6.73%)	
Salvage	226 (0.18%)	310 (1.03%)	
Cross-clamp Time	76.46 (41.56)	84.29 (51.21)	<0.001
Perfusion Time	107.17 (53.63)	123.61 (75.09)	<0.001

### Kaplan meier analysis

**Figure 3:**
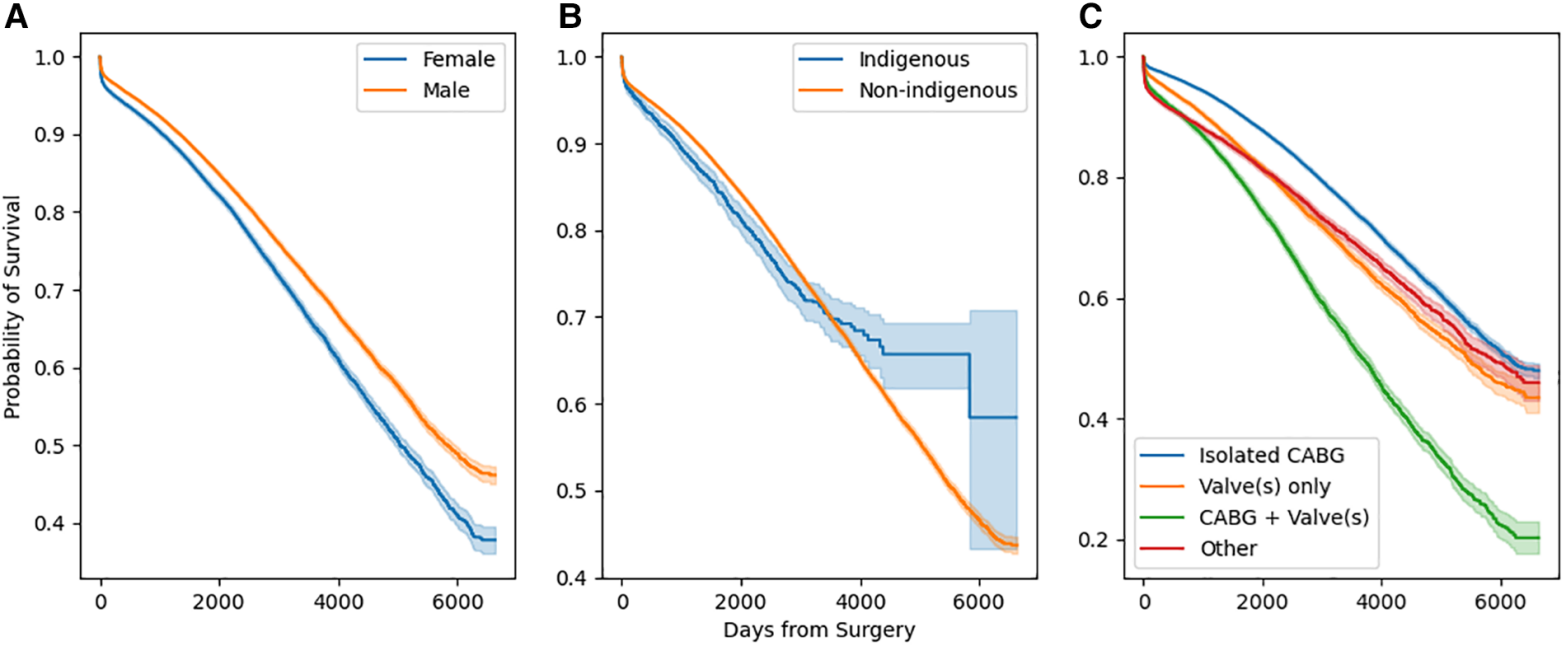
Stratified survival functions as defined by Kaplan-Meier estimators. Stratification by sex is depicted in (**a**), by race in (**b**), and by operation type in (**c**).

Stratified survival functions as estimated by the Kaplan-Meier estimator are shown in Figure 3. Women undergoing cardiac surgery had worse long-term survival. Indigenous people undergoing cardiac surgery had worse survival early after surgery, however, this effect reversed with very long-term (>10 years) survival. The combination of a coronary artery bypass and valve in the same operation conferred the worst long term mortality.

### Performance results

The best performing model was the GBM, with a C-index of 0.803 (0.002), compared with CPH modeling with 0.596 (0.042). The performance of the GBM on female patients was 0.800 (0.004) and for indigenous patients was 0.773 (0.012). All performance scores for each benchmarked model, including their bias assessment are reported in Table 2.

**Table 2:** Performance and bias Assessment.

Model	C-index (Standard Deviation)	C-index for Female Patients (Standard Deviation)	C-index for Indigenous Patients (Standard Deviation)
Cox Proportion Hazards Model	0.596 (0.042)	0.593 (0.039)	0.590 (0.044)
Decision Tree	0.729 (0.014)	0.721 (0.015)	0.677 (0.033)
Random Forest	0.791 (0.003)	0.789 (0.004)	0.774 (0.008)
Gradient Boosting Machine	0.803 (0.002)	0.800 (0.004)	0.773 (0.012)

### Feature importance

As gradient boosting machines had the highest performance, feature importance was extracted from its weights. Table 3 reports the top 15 most important variables along with their feature importance score (higher is more significant). Age was the most significant risk factor, followed by operation type, length of hospital stay, and drain output in the first 4 h.

**Table 3:** Top 15 most important features. A higher value indicates a more important feature for predicting time to mortality.

Feature	Score
Age	0.43
Type of Procedure	0.15
Length of Stay	0.13
Drain output in first 4 h (ml)	0.03
Inotrope >4 h postoperatively	0.03
Clopidogrel within 7 days of surgery	0.02
Perfusion Time	0.02
Length of Intubation (Hours)	0.02
Cross-clamp Time	0.01
NYHA Classification Class I	0.01
Highest Postoperative Creatinine	0.01
Discharge Destination (Home)	0.01
Lowest Postoperative Haemaglobin	0.01
Length of ICU Stay (Hours)	0.01
Preoperative Creatinine	0.01

## Discussion

This is the first study to investigate tree-based time-to-event models to predict survival. It is also the first to study machine learning bias in this context. Our results show that machine learning outperforms the current gold standard in medical research, the Cox proportional hazards model. Additionally, we have shown that high performing machine learning approaches can be used to determine the most important features for predicting long-term mortality.

### Interpretation of univariate analysis

The initial data exploration included a univariate analysis with a Kaplan-Meier estimator, which indicated sex differences in long-term mortality. The finding that there is an association between female sex and higher long-term mortality has been previously identified (21). Women who undergo cardiac surgery tend to be older, have greater comorbities, and need emergency or urgent surgery (22, 23). The existing literature on whether female sex is an independent risk factor for long-term mortality is mixed with analyses usually conducted using linear modeling (21, 24). Sex was not ranked as a significant variable in the gradient boosting model, which adds evidence to the argument that the sex difference is mostly explained by other covariates associated with female sex.

Indigenous mortality is less studied, however, a recent analysis of patients undergoing coronary artery bypass grafting found they were younger but suffering from a higher prevalence of comorbidities (25). As age is the most predictive feature for mortality, the younger indigenous cohort could account for later equalization and gain in survival for these patients.

### Effectiveness of machine learning

Similar to the pre-existing literature of tree-based methods in cardiac surgery, GBM are often as good or better than pre-existing methods (4, 26). The effectiveness of these GBM and RF models in healthcare has been shown across many domains and forms the basis of many clinical support tools (27, 28). The basis for the effectiveness of these models has recently been empirically determined with several attractive inductive biases of tree-based models. They are robust to uninformative features, preserve dataset orientation, and capable of learning non-smooth functions (11). This is in contrast to linear models, such as CPH, that do not share these properties.

An important feature of any predictive model in healthcare is explainability (29). We have demonstrated that a trained model can provide global feature importance which provides insight into risk factors that may have been previously overlooked. One interesting predictive feature the GBM learned was clopidogrel use within 7 days preoperatively. While recent evidence does not find that clopidogrel use impacted short or long-term mortality, this analysis was performed using CPH modeling (30). The predictive importance of clopidogrel in this analysis is interesting, however, it serves to underscore the importance of novel ML methods for hypothesis-generating research.

### Clinical impact of tree-based ML

The impact of ML in the clinical domain has been slow, however, tree-based risk stratification tools and apps, such as predictive optimal trees in emergency surgery (POTTER), have introduced clinicians to their routine use (7). Further potential exists for these models to provide automated feedback and risk modeling in electronic medical systems. Tree-based survival analysis could provide additional depth to modeling in both clinician facing and automated systems.

## Limitations

There are a number of limitations to our study. Firstly, we made the assumption that data was either missing completely at random, or missing at random. While we believe this is a safe assumption, there may be instances where data is missing not at random that biases our imputation approach. Additionally, compared to CPH and decision tree methods, RF and GBM were significantly more computationally expensive. In settings with very large datasets and limited computational resources they may not be appropriate choices. The use of less computationally intensive algorithms, such as light gradient boosting machine, could improve computational performance however the gain in this analysis would be marginal (31). Furthermore, feature importance rankings derived from GBM or RF do not provide a direction. In CPH models, the coefficients are easily interpretable and provide a direction for the features effect. Where this is important, alternative explanatory methods should be considered. Finally, in our bias analysis, while there was no difference in performance for female sex, there was a noticeable but small drop in performance for the indigenous patients. Where these models are used in clinical settings, consideration should be made to ensure separate machine learning models are trained for this important cohort to address the potential for bias (32).

## Conclusions

Tree-based learning for survival analysis is a non-parametric and performant alternative to Cox proportional hazards modeling. Within the tree-based learning methods, gradient boosting machines perform the best as measured by the C-index. These models can provide risk profiles to guide clinical reasoning and uncover new questions for future research.

## Data Availability

The datasets presented in this article are not readily available because The ANZSCTS National Audit Database contains confidential patient information and is therefore not publicly available. It is accessible through application to the ANZSCTS Database research committee. Requests to access the datasets should be directed to https://anzscts.org/database/. Further enquiries can be directed to the corresponding author.
