# Supplementary material for: Tree-based survival analysis improves mortality prediction in cardiac surgery
Source: Front Cardiovasc Med. 2023 Jul 10;10:1211600. doi: 10.3389/fcvm.2023.1211600 (PMC10365268; doi:10.3389/fcvm.2023.1211600)
Supplement: Supplementary file 1 [file Datasheet1.docx]

**Appendix S1:** Hyperparameters and Search Ranges

Cox Proportional Hazards with Elastic Net Penalty:

- 'l1_ratio':{"max":1.0,"min":0.0,"distribution":"uniform"}

Decision Tree

- 'max_depth':{"max":12,"min":2,"distribution":"int_uniform"},
- 'min_samples_split':{"max":100,"min":1,"distribution":"int_uniform"},
- 'min_samples_leaf':{"max":199,"min":1,"distribution":"int_uniform"}

Random Forest

- 'max_depth':{"max":12,"min":2,"distribution":"int_uniform"},
- 'min_samples_split':{"max":100,"min":2,"distribution":"int_uniform"},
- 'min_samples_leaf':{"max":100,"min":1,"distribution":"int_uniform"},
- 'max_samples':{"max":1.0,"min":0.0,"distribution":"uniform"},

Gradient Boosting Machines

- 'learning_rate':{"max":1.0,"min":0.0001,"distribution":"log_uniform_values"},
- 'max_depth':{"max":12,"min":2,"distribution":"int_uniform"},
- 'min_samples_split':{"max":100,"min":1,"distribution":"int_uniform"},
- 'min_samples_leaf':{"max":100,"min":1,"distribution":"int_uniform"},
- 'subsample':{"max":1.0,"min":0.0,"distribution":"uniform"},

**Table S1:** Patient Characteristics for Continuous Variables

| **Variable** | **Survived Mean (SD)** | **Mortality Mean (SD)** | **p-value** |
| --- | --- | --- | --- |
| Length of ICU Stay (Hours) | 64.59 (91.89) | 102.50 (91.89) | <0.001 |
| Length of Intubation (Hours) | 19.30 (59.69) | 42.75 (59.69) | <0.001 |
| Operation number | 0.00 (0.00) | 0.18 (0.00) | <0.001 |
| Number of procedures on the same day | 1.04 (0.21) | 1.05 (0.21) | <0.001 |
| Age | 64.28 (12.87) | 71.05 (12.87) | <0.001 |
| Preoperative Creatinine | 95.03 (67.15) | 120.67 (67.15) | <0.001 |
| Preoperative Haemaglobin | 135.66 (18.72) | 123.14 (18.72) | <0.001 |
| Last dose of aspirin | 0.86 (1.53) | 0.79 (1.53) | 0.13 |
| Height | 170.02 (10.23) | 167.77 (10.23) | <0.001 |
| Weight | 83.04 (17.88) | 79.17 (17.88) | <0.001 |
| Ejection Fraction | 56.19 (29.53) | 51.19 (29.53) | <0.001 |
| BMI | 28.77 (8.03) | 28.25 (8.03) | <0.001 |
| BSA | 1.90 (0.22) | 1.84 (0.22) | <0.001 |
| eGFR | 88.75 (36.32) | 67.45 (36.32) | <0.001 |
| Cross-clamp Time | 76.46 (41.56) | 84.29 (41.56) | <0.001 |
| Perfusion Time | 107.17 (53.63) | 123.61 (53.63) | <0.001 |
| Minimum intraoperative haemaglobin | 114.79 (141.73) | 111.85 (141.73) | 0.38 |
| Units of RBC | 3.50 (6.23) | 5.75 (6.23) | <0.001 |
| Units of Platelets Transfused | 2.07 (4.16) | 2.64 (4.16) | <0.001 |
| Milligrams of Novo7 | 0.34 (39.47) | 0.23 (39.47) | 0.77 |
| Units of Cryoprecipitate | 2.92 (9.93) | 3.48 (9.93) | <0.001 |
| Units of FFP | 2.18 (3.59) | 3.70 (3.59) | <0.001 |
| Drain output in first 4 hours (mL) | 249.83 (243.23) | 309.45 (243.23) | <0.001 |
| Highest Postoperative Creatinine | 115.69 (88.47) | 167.56 (88.47) | <0.001 |
| Lowest Postoperative Haemaglobin | 91.52 (17.35) | 83.52 (17.35) | <0.001 |

**Table S2: Patient Characteristics for Categorical Variables**

| Variable | Survived Counts | Mortality Counts | p-value |
| --- | --- | --- | --- |
| Type of Procedure | | | |
| - Isolated CABG  - Valve(s) only  - Other  - Valve(s) + CABG | 67880 (54.85%)  25429 (20.55%)  19594 (15.83%)  10690 (8.64%) | 14290 (47.33%)  6040 (20.00%)  4996 (16.55%)  4797 (15.89%) | <0.001 |
| Redo operation | | | |
| - No  - Yes | 114878 (92.83%)  8872 (7.17%) | 26608 (88.12%)  3586 (11.88%) | <0.001 |
| Sex | | | |
| - Male  - Female | 91839 (74.21%)  31911 (25.79%) | 20844 (69.03%)  9350 (30.97%) | <0.001 |
| Indigenous | | | |
| - No  - Yes | 118921 (96.10%)  3325 (2.69%) | 29206 (96.73%)  665 (2.20%) | <0.001 |
| Elective Procedure | | | |
| - No  - Yes | 88552 (71.56%)  35151 (28.40%) | 21747 (72.02%)  8437 (27.94%) | 0.11 |
| Insurance Status | | | |
| - Medicare  - Private  - DVA  - 7.0  - Overseas  - Other  - Self-insured | 85455 (69.05%)  33128 (26.77%)  1235 (1.00%)  962 (0.78%)  733 (0.59%)  482 (0.39%)  413 (0.33%) | 22800 (75.51%)  5559 (18.41%)  793 (2.63%)  189 (0.63%)  58 (0.19%)  54 (0.18%)  49 (0.16%) | <0.001 |
| Smoking History | | | |
| - Yes  - No | 69496 (56.16%)  52670 (42.56%) | 18646 (61.75%)  11258 (37.29%) | <0.001 |
| Current Smoker | | | |
| - No  - Yes | 53015 (42.84%)  17172 (13.88%) | 15462 (51.21%)  3644 (12.07%) | <0.001 |
| Diabetes | | | |
| - No  - Yes | 89276 (72.14%)  34295 (27.71%) | 19552 (64.75%)  10557 (34.96%) | <0.001 |
| Diabetes Control | | | |
| - Oral  - Insulin  - Diet  - None | 19590 (15.83%)  9201 (7.44%)  4730 (3.82%)  1072 (0.87%) | 5582 (18.49%)  3281 (10.87%)  1526 (5.05%)  316 (1.05%) | <0.001 |
| High Cholesterol | | | |
| - Yes  - No | 81269 (65.67%)  42288 (34.17%) | 20265 (67.12%)  9827 (32.55%) | <0.001 |
| Peroperative Dialysis Requirement | | | |
| - No  - Yes | 122220 (98.76%)  1361 (1.10%) | 28951 (95.88%)  1177 (3.90%) | <0.001 |
| History of Renal Transplant | | | |
| - 0.0  - Yes | 107754 (87.07%)  483 (0.39%) | 18159 (60.14%)  147 (0.49%) | <0.001 |
| Hypertension | | | |
| - Yes  - No | 87245 (70.50%)  36319 (29.35%) | 23515 (77.88%)  6589 (21.82%) | <0.001 |
| Cerebrovascular Disease | | | |
| - No  - Yes | 112221 (90.68%)  11344 (9.17%) | 25080 (83.06%)  5027 (16.65%) | <0.001 |
| Type of Cerebrovascular Disease | | | |
| - CVA  - RIND/TIA  - Carotid Test  - Coma | 5588 (4.52%)  3875 (3.13%)  1802 (1.46%)  36 (0.03%) | 2528 (8.37%)  1557 (5.16%)  868 (2.87%)  29 (0.10%) | <0.001 |
| Carotid Stenosis (>50%) | | | |
| - 0.0  - 1.0 | 41488 (33.53%)  4856 (3.92%) | 1952 (6.46%)  268 (0.89%) | 0.02 |
| Peripheral Vascular Disease | | | |
| - No  - Yes | 114849 (92.81%)  8719 (7.05%) | 25098 (83.12%)  5001 (16.56%) | <0.001 |
| Lung Disease | | | |
| - No  - Yes | 108687 (87.83%)  14885 (12.03%) | 23877 (79.08%)  6236 (20.65%) | <0.001 |
| Lung Disease Type | | | |
| - Mild  - Moderate  - Severe | 10693 (8.64%)  3138 (2.54%)  1040 (0.84%) | 3947 (13.07%)  1646 (5.45%)  636 (2.11%) | <0.001 |
| Infective Endocarditis | | | |
| - No  - Yes | 120143 (97.09%)  3420 (2.76%) | 29026 (96.13%)  1075 (3.56%) | <0.001 |
| Immunosuppression | | | |
| - No  - Yes | 120693 (97.53%)  2869 (2.32%) | 28838 (95.51%)  1267 (4.20%) | <0.001 |
| Previous Myocardial Infarction | | | |
| - No  - Yes | 82048 (66.30%)  41544 (33.57%) | 17639 (58.42%)  12483 (41.34%) | <0.001 |
| Previous Myocardial Infarction Type | | | |
| - 1.0  - 2.0 | 25523 (20.62%)  7946 (6.42%) | 4885 (16.18%)  1515 (5.02%) | 0.92 |
| Timeframe of Previous Myocardial Infarction | | | |
| - >21 days  - >7 - 21 days  - 1-7 days  - >6 hours - <24 hours  - <=6 hours | 17043 (13.77%)  11848 (9.57%)  11145 (9.01%)  933 (0.75%)  541 (0.44%) | 6341 (21.00%)  3027 (10.03%)  2488 (8.24%)  367 (1.22%)  239 (0.79%) | <0.001 |
| CCS Angina Classification | | | |
| - 0.0  - 2.0  - 3.0  - 4.0  - 1.0 | 51353 (41.50%)  29307 (23.68%)  17463 (14.11%)  13092 (10.58%)  11994 (9.69%) | 11235 (37.21%)  6683 (22.13%)  5214 (17.27%)  3975 (13.16%)  2821 (9.34%) | <0.001 |
| Preoperative IV GTN | | | |
| - No  - Yes | 67711 (54.72%)  4686 (3.79%) | 17076 (56.55%)  1908 (6.32%) | <0.001 |
| Preoperative IV Heparin | | | |
| - No  - Yes | 59950 (48.44%)  12499 (10.10%) | 15395 (50.99%)  3612 (11.96%) | <0.001 |
| Preoperative Heparanoids | | | |
| - No  - Yes | 66991 (54.13%)  5456 (4.41%) | 17082 (56.57%)  1928 (6.39%) | <0.001 |
| Congestive Heart Failure | | | |
| - No  - Yes | 101910 (82.35%)  21663 (17.51%) | 19511 (64.62%)  10602 (35.11%) | <0.001 |
| Congestive Heart Failure at Current Admission | | | |
| - No  - Yes | 13729 (11.09%)  9650 (7.80%) | 6023 (19.95%)  5408 (17.91%) | <0.001 |
| NYHA Class | | | |
| - I  - II  - III  - IV | 48427 (39.13%)  43970 (35.53%)  23351 (18.87%)  5292 (4.28%) | 9195 (30.45%)  8528 (28.24%)  7911 (26.20%)  3236 (10.72%) | <0.001 |
| Preoperative Shock | | | |
| - No  - Yes | 121759 (98.39%)  1813 (1.47%) | 28699 (95.05%)  1422 (4.71%) | <0.001 |
| Preoperative Resuscitation | | | |
| - No  - Yes | 122662 (99.12%)  908 (0.73%) | 29482 (97.64%)  634 (2.10%) | <0.001 |
| Preoperative Arrhythmia | | | |
| - No  - Yes | 104539 (84.48%)  19013 (15.36%) | 22727 (75.27%)  7376 (24.43%) | <0.001 |
| Preoperative AF | | | |
| - No  - Yes | 15620 (12.62%)  14923 (12.06%) | 9758 (32.32%)  6045 (20.02%) | <0.001 |
| Preoperative AF Type | | | |
| - 1.0  - 3.0 | 7238 (5.85%)  6365 (5.14%) | 2207 (7.31%)  1712 (5.67%) | <0.001 |
| Preoperative Heart Block | | | |
| - No  - Yes | 28866 (23.33%)  1559 (1.26%) | 14942 (49.49%)  742 (2.46%) | 0.07 |
| Preoperative Ventricular Arrhythmia | | | |
| - No  - Yes | 28542 (23.06%)  1886 (1.52%) | 14940 (49.48%)  739 (2.45%) | <0.001 |
| Preoperative Arrhythmia Other | | | |
| - No  - Yes | 29847 (24.12%)  568 (0.46%) | 15464 (51.22%)  206 (0.68%) | <0.001 |
| Preoperative PPM | | | |
| - No  - Yes | 104212 (84.21%)  3168 (2.56%) | 16681 (55.25%)  1069 (3.54%) | <0.001 |
| Preoperative Inotropes on day of surgery | | | |
| - No  - Yes | 120035 (97.00%)  3535 (2.86%) | 28524 (94.47%)  1591 (5.27%) | <0.001 |
| Preoperative Nitrates on day of surgery | | | |
| - No  - Yes | 118612 (95.85%)  4960 (4.01%) | 28353 (93.90%)  1757 (5.82%) | <0.001 |
| Anticoagulants <24hours before surgery | | | |
| - No  - Yes | 100956 (81.58%)  22585 (18.25%) | 23593 (78.14%)  6509 (21.56%) | <0.001 |
| Systemic Steroids <24hours before surgery | | | |
| - No  - Yes | 120565 (97.43%)  2054 (1.66%) | 28821 (95.45%)  1167 (3.87%) | <0.001 |
| Aspirin within 7 days of surgery | | | |
| - Yes  - No  - 9.0 | 66887 (54.05%)  51198 (41.37%)  393 (0.32%) | 12361 (40.94%)  11862 (39.29%)  62 (0.21%) | <0.001 |
| Clopidogrel within 7 days of surgery | | | |
| - No  - Yes | 106288 (85.89%)  12187 (9.85%) | 21510 (71.24%)  2777 (9.20%) | <0.001 |
| Ticagrelor within 7 days of surgery | | | |
| - 0.0  - 1.0 | 43611 (35.24%)  2987 (2.41%) | 2128 (7.05%)  105 (0.35%) | 0.00 |
| Glycoprotein inhibtor within 7 days of surgery | | | |
| - No  - Yes | 117044 (94.58%)  1167 (0.94%) | 23817 (78.88%)  323 (1.07%) | <0.001 |
| Abciximab within 7 days of surgery | | | |
| - No  - Yes | 118122 (95.45%)  326 (0.26%) | 24162 (80.02%)  104 (0.34%) | <0.001 |
| Other antiplatelet within 7 days of surgery | | | |
| - No  - Yes | 113591 (91.79%)  4677 (3.78%) | 23361 (77.37%)  826 (2.74%) | <0.001 |
| Previous CTS intervention | | | |
| - No  - Yes | 99883 (80.71%)  23703 (19.15%) | 23480 (77.76%)  6651 (22.03%) | <0.001 |
| Transluminal coronary intervention before surgery | | | |
| - Yes  - No | 14522 (11.73%)  8254 (6.67%) | 4455 (14.75%)  3256 (10.78%) | <0.001 |
| Previous PTCA or Stent | | | |
| - 0.0  - 1.0 | 13171 (10.64%)  872 (0.70%) | 2173 (7.20%)  313 (1.04%) | <0.001 |
| Previous Angiography | | | |
| - Yes  - No | 111695 (90.26%)  11802 (9.54%) | 26920 (89.16%)  3153 (10.44%) | <0.001 |
| Ejection Fraction Estimate | | | |
| - Normal >60%  - Mild 46-60%  - Mod 30-45%  - Severe <30% | 64692 (52.28%)  37549 (30.34%)  14273 (11.53%)  4364 (3.53%) | 12569 (41.63%)  8587 (28.44%)  5489 (18.18%)  2563 (8.49%) | <0.001 |
| Left main disease | | | |
| - No  - Yes | 103598 (83.72%)  19741 (15.95%) | 24391 (80.78%)  5534 (18.33%) | <0.001 |
| Number of diseased coronary systems | | | |
| - three  - none  - two  - one | 54047 (43.67%)  38570 (31.17%)  20995 (16.97%)  9659 (7.81%) | 14002 (46.37%)  8187 (27.11%)  4941 (16.36%)  2771 (9.18%) | <0.001 |
| Urgency | | | |
| - Elective  - Urgent  - Emergency  - Salvage | 87018 (70.32%)  31667 (25.59%)  4755 (3.84%)  226 (0.18%) | 18707 (61.96%)  9127 (30.23%)  2033 (6.73%)  310 (1.03%) | <0.001 |
| Direct from Cath Lab | | | |
| - No  - Yes | 122170 (98.72%)  1210 (0.98%) | 29505 (97.72%)  514 (1.70%) | <0.001 |
| CAGB | | | |
| - Yes  - No | 82895 (66.99%)  40771 (32.95%) | 20826 (68.97%)  9348 (30.96%) | <0.001 |
| Valve Operation | | | |
| - No  - Yes | 76717 (61.99%)  46936 (37.93%) | 16905 (55.99%)  13260 (43.92%) | <0.001 |
| Other Procedure | | | |
| - No  - Yes | 108860 (87.97%)  14798 (11.96%) | 26609 (88.13%)  3557 (11.78%) | 0.40 |
| Transplant | | | |
| - No  - Yes | 26339 (21.28%)  938 (0.76%) | 13028 (43.15%)  362 (1.20%) | <0.001 |
| Congenital or Other | | | |
| - No  - Yes | 26090 (21.08%)  1211 (0.98%) | 13306 (44.07%)  84 (0.28%) | <0.001 |
| Minimally invasive | | | |
| - No  - Yes | 118444 (95.71%)  5018 (4.05%) | 29465 (97.59%)  600 (1.99%) | <0.001 |
| Robot Assisted | | | |
| - no  - yes | 118115 (95.45%)  403 (0.33%) | 24287 (80.44%)  24 (0.08%) | <0.001 |
| On-pump | | | |
| - Yes  - No | 116018 (93.75%)  7652 (6.18%) | 28143 (93.21%)  2029 (6.72%) | <0.001 |
| Cardioplegia | | | |
| - Yes  - No | 112148 (90.62%)  4197 (3.39%) | 26939 (89.22%)  1460 (4.84%) | <0.001 |
| Cardioplegia Type | | | |
| - 1.0  - 2.0  - 3.0 | 37845 (30.58%)  2316 (1.87%)  629 (0.51%) | 1588 (5.26%)  166 (0.55%)  31 (0.10%) | <0.001 |
| IABP at any time | | | |
| - No  - Yes | 119641 (96.68%)  3905 (3.16%) | 27581 (91.35%)  2552 (8.45%) | <0.001 |
| ECMO at any time | | | |
| - 0.0  - 1.0 | 46375 (37.47%)  208 (0.17%) | 2025 (6.71%)  208 (0.69%) | <0.001 |
| VAD at any time | | | |
| - No  - Yes | 46562 (37.63%)  211 (0.17%) | 2189 (7.25%)  53 (0.18%) | <0.001 |
| Intraoperative antifibrinolytic | | | |
| - yes  - no  - 9.0 | 87577 (70.77%)  19606 (15.84%)  718 (0.58%) | 14314 (47.41%)  3755 (12.44%)  105 (0.35%) | <0.001 |
| Antifibrinolytic type | | | |
| - tranexamic acid  - other  - 4.0  - trasylol | 82929 (67.01%)  3907 (3.16%)  474 (0.38%)  268 (0.22%) | 13144 (43.53%)  1028 (3.40%)  118 (0.39%)  25 (0.08%) | <0.001 |
| RBC transfusion | | | |
| - No  - Yes | 83098 (67.15%)  40480 (32.71%) | 17341 (57.43%)  12768 (42.29%) | <0.001 |
| Other blood products transfused | | | |
| - No  - Yes | 93711 (75.73%)  29860 (24.13%) | 18880 (62.53%)  11220 (37.16%) | <0.001 |
| Readmission to ICU | | | |
| - No  - Yes | 119599 (96.65%)  3222 (2.60%) | 27657 (91.60%)  1929 (6.39%) | <0.001 |
| Reintubation | | | |
| - No  - Yes | 106420 (86.00%)  2221 (1.79%) | 16489 (54.61%)  1510 (5.00%) | <0.001 |
| Return to Theatre | | | |
| - No  - Yes | 115660 (93.46%)  7944 (6.42%) | 25943 (85.92%)  3898 (12.91%) | <0.001 |
| Postoperative AKI | | | |
| - No  - Yes | 119172 (96.30%)  4421 (3.57%) | 26548 (87.92%)  3285 (10.88%) | <0.001 |
| Postoperative MI | | | |
| - No  - Yes | 122905 (99.32%)  641 (0.52%) | 29378 (97.30%)  384 (1.27%) | <0.001 |
| Postoperative Shock | | | |
| - No  - Yes | 103230 (83.42%)  2558 (2.07%) | 15500 (51.33%)  1636 (5.42%) | <0.001 |
| Inotrope >4 hours postoperatively | | | |
| - 0.0  - 1.0 | 63321 (51.17%)  55025 (44.46%) | 13394 (44.36%)  10510 (34.81%) | <0.001 |
| Inotrope for low cardiac output | | | |
| - No  - Yes | 96030 (77.60%)  21434 (17.32%) | 15806 (52.35%)  8045 (26.64%) | <0.001 |
| Vasopressor for low SVR | | | |
| - No  - Yes | 88878 (71.82%)  28585 (23.10%) | 17777 (58.88%)  6076 (20.12%) | <0.001 |
| New postoperative arrhythmia | | | |
| - No  - Yes | 89919 (72.66%)  33674 (27.21%) | 19020 (62.99%)  10831 (35.87%) | <0.001 |
| New postoperative heart block | | | |
| - No  - Yes | 33035 (26.69%)  1789 (1.45%) | 11043 (36.57%)  518 (1.72%) | 0.01 |
| New postoperative bradyarrhythmia | | | |
| - No  - Yes | 33684 (27.22%)  1130 (0.91%) | 11103 (36.77%)  457 (1.51%) | <0.001 |
| Postoperative cardiac arrest | | | |
| - No  - Yes | 33959 (27.44%)  852 (0.69%) | 10344 (34.26%)  1216 (4.03%) | <0.001 |
| New postoperative AF | | | |
| - Yes  - No | 29930 (24.19%)  4893 (3.95%) | 9121 (30.21%)  2435 (8.06%) | <0.001 |
| New postoperative ventricular arrhythmia | | | |
| - No  - Yes | 32660 (26.39%)  1979 (1.60%) | 10517 (34.83%)  971 (3.22%) | <0.001 |
| Postoperative stroke | | | |
| - No  - Yes | 122229 (98.77%)  1185 (0.96%) | 28737 (95.17%)  1007 (3.34%) | <0.001 |
| Postoperative TIA | | | |
| - No  - Yes | 122859 (99.28%)  554 (0.45%) | 29504 (97.71%)  264 (0.87%) | <0.001 |
| Postoperative Coma | | | |
| - No  - Yes | 123214 (99.57%)  198 (0.16%) | 29312 (97.08%)  432 (1.43%) | <0.001 |
| Postoperative Pulmonary Embolism | | | |
| - No  - Yes | 123136 (99.50%)  274 (0.22%) | 29606 (98.05%)  138 (0.46%) | <0.001 |
| Postoperative Chest Infection | | | |
| - No  - Yes | 118788 (95.99%)  4806 (3.88%) | 27416 (90.80%)  2416 (8.00%) | <0.001 |
| Deep sternal wound infection | | | |
| - No  - Yes | 122999 (99.39%)  598 (0.48%) | 29482 (97.64%)  371 (1.23%) | <0.001 |
| Superficial skin infection | | | |
| - 0.0  - 1.0 | 45975 (37.15%)  622 (0.50%) | 2075 (6.87%)  44 (0.15%) | 0.01 |
| Deep Skin Infection | | | |
| - 0.0  - 1.0 | 46513 (37.59%)  84 (0.07%) | 2103 (6.96%)  16 (0.05%) | <0.001 |
| Thoracotomy infection | | | |
| - No  - Yes | 123529 (99.82%)  67 (0.05%) | 29793 (98.67%)  60 (0.20%) | <0.001 |
| Postoperative Aortic Dissection | | | |
| - No  - Yes | 123309 (99.64%)  65 (0.05%) | 29685 (98.31%)  43 (0.14%) | <0.001 |
| Postoperative Limb Ischaemia | | | |
| - No  - 3.0  - 2.0  - Yes | 123092 (99.47%)  88 (0.07%)  82 (0.07%)  34 (0.03%) | 29477 (97.63%)  95 (0.31%)  85 (0.28%)  51 (0.17%) | <0.001 |
| Postoperative bleeding | | | |
| - No  - Yes | 122614 (99.08%)  759 (0.61%) | 29200 (96.71%)  529 (1.75%) | <0.001 |
| Postoperative GIT complication | | | |
| - No  - Yes | 122014 (98.60%)  1359 (1.10%) | 28591 (94.69%)  1139 (3.77%) | <0.001 |
| Postoperative multisystem failure | | | |
| - No  - Yes | 122911 (99.32%)  463 (0.37%) | 28046 (92.89%)  1685 (5.58%) | <0.001 |
| Discharge location | | | |
| - home  - rehabilitation unit/hospital  - hospital in the home  - local/referring hospital  - hospital mortality  - 6.0 | 93626 (75.66%)  11300 (9.13%)  7824 (6.32%)  5770 (4.66%)  48 (0.04%)  3 (0.00%) | 12959 (42.92%)  4234 (14.02%)  3907 (12.94%)  2352 (7.79%)  1325 (4.39%)  8 (0.03%) | <0.001 |
| Elected for Withdrawal of Treatment | | | |
| - No  - yes | 117195 (94.70%)  174 (0.14%) | 28510 (94.42%)  120 (0.40%) | <0.001 |
